# Pituitary Apoplexy in a Non-Functioning PitNET After Cabergoline Use: Case Report and Review of the Literature

**DOI:** 10.3390/jcm14145089

**Published:** 2025-07-17

**Authors:** Federica De Luca, Margherita Paccagnella, Anna Pizzo, Giulia Zuolo, Veronica Calabrò, Stella Bernardi

**Affiliations:** 1 Department of Medical Surgical and Health Sciences, University of Trieste, Cattinara Teaching Hospital, Strada di Fiume 447, 34149 Trieste, Italy; federica.deluca@studenti.units.it (F.D.L.); margherita.paccagnella@studenti.units.it (M.P.); anna.pizzo@studenti.units.it (A.P.); 2 Endocrinology Unit Medicina Clinica (ASUGI), Cattinara Teaching Hospital, Strada di Fiume 447, 34149 Trieste, Italy; giulia.zuolo@asugi.sanita.fvg.it (G.Z.); veronica.calabro@asugi.sanita.fvg.it (V.C.)

**Keywords:** pituitary apoplexy, cabergoline, dopamine agonist, PitNET, non-functioning PitNET, NFPA

## Abstract

**Background/Objectives**: Pituitary apoplexy (PA) is a rare medical emergency characterized by the sudden onset of symptoms resulting from hemorrhage and/or infarction within the pituitary gland. Precipitating factors include the use of dopamine agonists (DAs), whose main indication is the treatment of prolactin (PRL)-secreting pituitary neuroendocrine tumors (PitNETs), but which can also be considered in non-functioning PitNETs. Here we report a case of PA in a patient taking cabergoline for a non-functioning PitNET, followed by a review of the literature focusing on the cases of PA associated with the use of DAs. **Methods**: A review of the literature was performed, searching Pubmed for other clinical cases of PA associated with the use of DAs, from inception to March 2025. **Results**: We found 43 cases of PA associated with the use of DAs. All the patients had secreting tumors: 86% were classified as PRL-secreting PitNETs, 7% were classified as GH-secreting PitNETs, and 4.6% included a mixed PRL/GH-secreting PitNET and a TSH-secreting PitNET. By contrast, here we present a case of PA in a non-functioning PitNET during cabergoline therapy. Our patient was managed conservatively and endocrine function recovered spontaneously. In our case, cabergoline might have promoted PA, which is consistent with the reported efficacy of cabergoline in inducing tumor shrinkage of non-functioning PitNETs that express dopamine 2 receptors, including silent PIT1 and SF1 or NULL tumors. **Conclusions**: Our case confirms cabergoline efficacy in non-functioning PitNETs and sheds light on a possible complication of its use. Patients, particularly those with large tumors, should be closely monitored for this occurrence.

## 1. Introduction

Pituitary apoplexy (PA) is a rare medical emergency characterized by the sudden onset of symptoms resulting from a hemorrhage and/or infarction within the pituitary gland. In 1898, Bailey recorded the first occurrence of bleeding linked to a pituitary tumor [1], but it was not until 1950 that the term “pituitary apoplexy” was introduced [2], meaning “a copious extravasation of blood into the pituitary”. Clinically, this condition arises abruptly with severe headaches sometimes associated with nausea, vomiting, visual impairments, and varying levels of anterior pituitary hormone deficiency. Involvement of the corticotropic axis can lead to pronounced hypotension and acute hyponatremia, which compromise consciousness [3,4].

PA usually occurs in patients with a pre-existing macro-PitNET (pituitary neuroendocrine tumor), whose commonest subtype is represented by non-functioning PitNETs [3,5,6] followed by PRL-secreting PitNETs [7,8]; however, normal pituitary glands can also be affected. Precipitating factors are present in 10–40% of cases [3] and they include not only major surgery, radiotherapy, pregnancy, anticoagulant therapy, and coagulopathies [9,10], but also, and most importantly, the use of drugs influencing pituitary function, such as GnRH agonists, somatostatin analogues, and dopamine agonists (DAs), namely bromocriptine and cabergoline [4].

DAs represent the first-line treatment of PRL-secreting PitNETs, as they inhibit PRL secretion [11]. Nevertheless, they may also be considered as an off-label treatment in the management of other pituitary tumors expressing dopamine 2 receptors (D2-Rs), such as in non-functioning PitNETs, which remain the only pituitary tumor type for which no medical therapy has been approved yet. There is no universal consensus on their use in non-functioning PitNETs [12]. Some authors consider that a therapeutic trial with cabergoline is worthwhile because this therapy can effectively restrain growth or induce tumor shrinkage [12]. On the other hand, other authors maintain that the use of cabergoline needs more data to be considered an option to conventional therapies (radiotherapy or surgery) or surveillance [12].

In this paper, we report a case of PA in a patient taking cabergoline for a non-functioning PitNET. In order to provide more insights into DA-induced PA, the case vignette is followed by a review of the literature reporting all the case reports/case series of DA-induced PA, their features, and outcomes.

## 2. Case Report of Pituitary Apoplexy in a Patient Taking Cabergoline for a Non-Functioning PitNET

**Figure 1:**
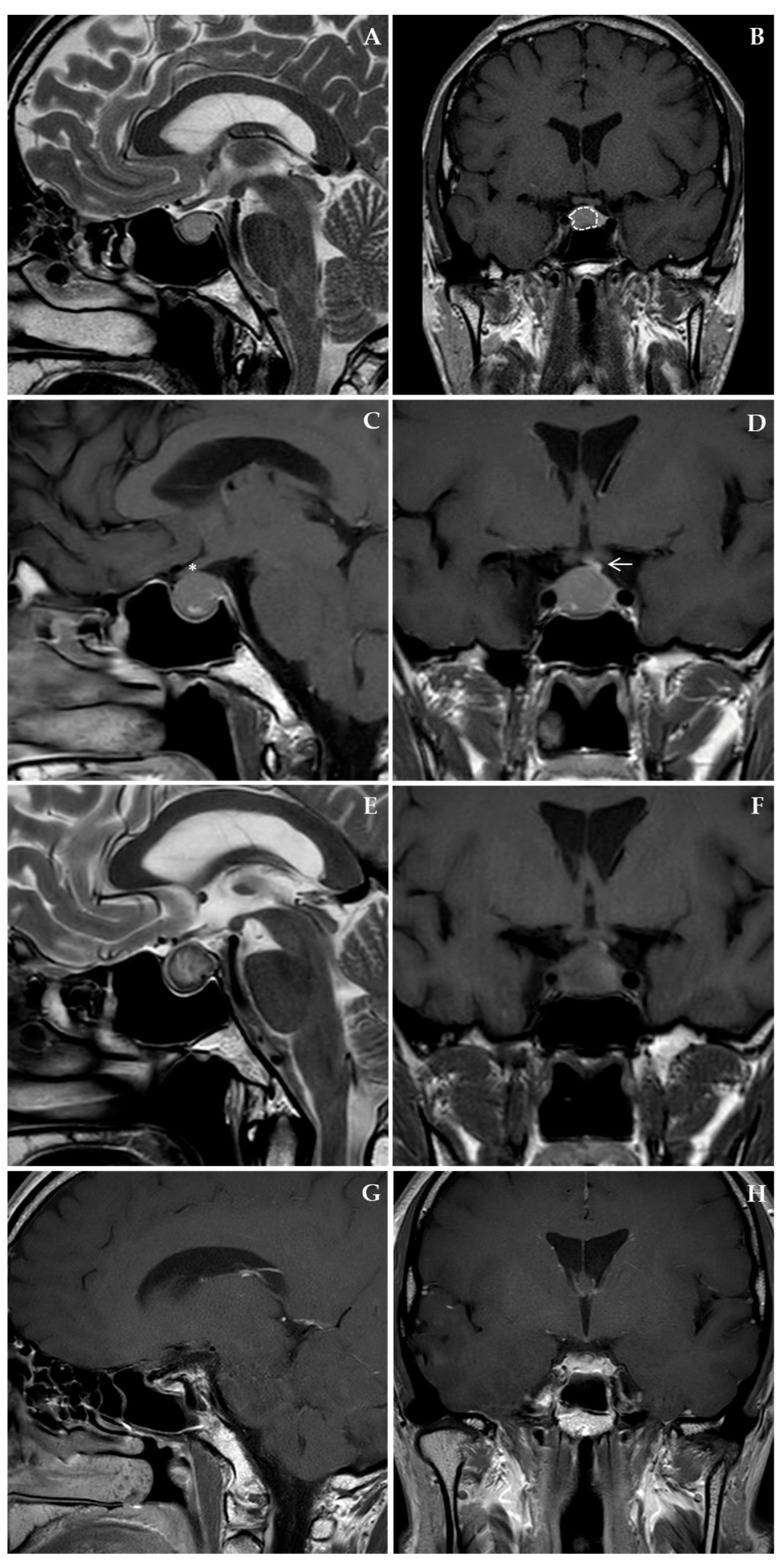
Brain MRI. (**A**) Sagittal T2-weighted images showing a macro-PitNET that is mildly hyperintense compared to the surrounding parenchyma. (**B**) Coronal T1-weighted contrast-enhanced images showing a macro-PitNET moderately hypointense compared to the surrounding parenchyma (dotted line). There is no evidence of residual healthy tissue between the lesion and the right cavernous sinus; the pituitary stalk appears deviated to the left, and the optic chiasm is within normal limits. (**C**) Sagittal and (**D**) coronal T1-weighted contrast-enhanced images showing an increase in the size of the macro-PitNET, with involvement of the right cavernous sinus, compression of the optic chiasm (asterisk), and persistence of pituitary stalk deviation (arrow). (**E**) Sagittal T2-weighted images and (**F**) coronal T1-weighted images contrast-enhanced, showing in the lateral left portion of the macro-PiNET a faint hyperintensity of the signal consistent with subacute bleeding. (**G**) Sagittal and (**H**) coronal T1-weighted contrast-enhanced images showing a marked reduction in the size of the adenohypophysis, with thin and peripheral enhancement post gadolinium.

A 38-year-old man presented to our Endocrine Service after a magnetic resonance imaging (MRI) of the brain, performed for recurrent headaches, showing a PitNET. This was an intrasellar lesion with a round morphology and well-defined margins, measuring 12 × 12 × 12 mm, that was located in the median and right paramedian position, causing a moderate mass effect on the pituitary stalk (Figure 1A,B). The patient had no past medical history and did not take any medication. On physical examination, he had no signs of hypercortisolism or acromegaly. Secondary sexual characteristics were normally represented, and he did not complain of erectile dysfunction. The laboratory exams showed a normal pituitary function (Table 1). The computerized static visual field study did not reveal any significant abnormalities. Based on the absence of any hormone hypersecretion, we diagnosed a clinically silent or non-functioning PitNET, and after consulting a neurosurgeon who did not indicate the need for pituitary surgery, we scheduled periodic follow-ups.

Consistent with the reported growth tendency of these tumors [13], the brain MRIs showed a slow progressive increase in the lesion, which increased to 10 × 10 × 20 mm after 2.5 years from presentation. At this stage, not only did the lesion deviate the pituitary stalk, but it also extended into the ipsilateral cavernous sinus and exerted a slight impression on the optic chiasm (Figure 1C,D). After multidisciplinary evaluation of the case, we decided not to perform surgery yet, and we started a therapeutic trial with cabergoline to restrain the growth of this progressing non-functioning PitNET [12]. Cabergoline was started at a dose of 0.5 mg once, then twice a week, and then progressively uptitrated to a final dose of 2 mg per week.

After five months, the patient presented to the ER department complaining of severe headache, vomiting, and fatigue. He received symptomatic treatment and was discharged on the same day after undergoing blood tests and a computed tomography scan showing an unmodified pituitary mass without signs of bleeding or venous thrombosis. One week later, given the symptom persistence, he came back to the ER department complaining of severe headache, vomiting, and fatigue. BP was 106/70 mmHg, HR was 55 bpm, and temperature was 38 °C. This time, laboratory exams showed hyponatremia (Na^+^ 118 mEq/L), normocytic anemia (Hb 11.9 g/dL, MCV 86.4 fl), and systemic inflammation (CRP 135.8 mg/L). This was associated with laboratory evidence of acute hypopituitarism (morning cortisol was 46.9 nmol/L, testosterone was undetectable, with low levels of LH and FSH), as shown in Table 1. A new MRI showed that the pituitary lesion was stable in size (15 × 15 × 18 mm), but in its left portion, there was an area of faint hyperintensity with a hypointense rim on T2-weighted images, consistent with a subacute hemorrhage (Figure 1E,F). Cabergoline was discontinued, and the patient was treated with saline and hydrocortisone (100 mg IV bolus, followed by hydrocortisone 100 mg IV every 12 h), as recommended in cases of adrenal crisis [14,15,16]. In the following days, hydrocortisone was gradually tapered and subsequently replaced with cortisone acetate 25 mg + 12.5 mg, and levothyroxine replacement therapy was introduced at the dose of 50 ug/day. Given the improvement in patient symptoms, laboratory exams, and the results of the brain MRI, the consulting neurosurgeon recommended only conservative management of the PA.

**Figure 2:**
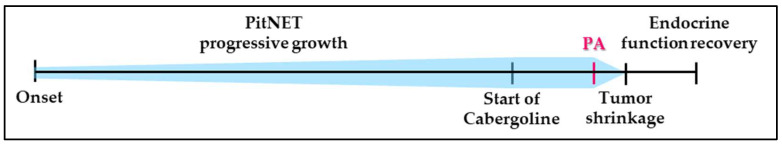
Timeline of the case report. This image summarizes the timing of main events: Cabergoline was started 2.5 years after presentation because of progressive growth of the pituitary neuroendocrine tumor (PitNET). Then, 5 months after cabergoline treatment initiation, the patient presented with pituitary apoplexy (PA). The patient was managed conservatively. After 2 months from PA, the magnetic resonance images showed tumor shrinkage, and after 6 months from PA, exams showed complete recovery of pituitary function.

Two weeks after discharge, the patient was well, and the hormonal workup showed ACTH 10.3 pg/mL, cortisol 236 nmol/L, fT4 7 pg/mL, TSH 0.28 µUI/mL, LH 1.0 mIU/mL, FSH 0.80 mIU/mL, and testosterone 0.5 ng/mL (Table 1). Cortisone acetate was reduced to 25 mg/day, and levothyroxine therapy was increased to 50 mcg 5 days/week and 75 mcg 2 days/week. Two months after discharge, a brain MRI was repeated, showing a marked reduction of the pituitary gland (Figure 1G,H). After four months, the low-dose ACTH stimulation test showed that cortisol was 231 nmol/L at 0 min, 435 nmol/L at 30 min, 430 nmol/L at 60 min, and 344 nmol/L at 90 min. The remaining blood tests documented fT4 8.8 pg/mL, TSH 0.54 µUI/mL, and testosterone 4.5 ng/mL (Table 1). The therapy with cortisone acetate was stopped, with instructions to take it only in case of an acute intercurrent illness, and levothyroxine was reduced to 25 mcg daily. After six months, the laboratory exams showed a complete recovery of pituitary function, and hormone replacement therapy was suspended (Table 1). The timeline of patient history is shown in Figure 2.

## 3. Review of the Cases of Pituitary Apoplexy in Patients Taking Dopamine Agonists

In order to review the features of PA in patients taking DAs, we analyzed all the studies reporting cases with an association between PA and therapy with cabergoline or bromocriptine. Our search was performed on Pubmed from inception to March 2025, as well as on previously published reviews of the literature [17,18,19,20]. The complete search string included the following keywords: “Pituitary Apoplexy” and “Cabergoline” or “Bromocriptine” or “Dopamine agonist”. We included case reports and case series with <10 patients. Original articles and reviews, as well as papers whose full text was not available, or papers written in languages other than English, were excluded. Studies were also excluded if they reported the wrong occurrence (e.g., aneurysm or herniation), or the wrong precipitating/risk factor, or multiple risk factors (e.g., chemotherapy with or without DAs). A total of 104 papers were retrieved, and at the end of our qualitative analysis, only 34 papers reporting 43 cases of PA were included in our literature review.

**Figure 3:**
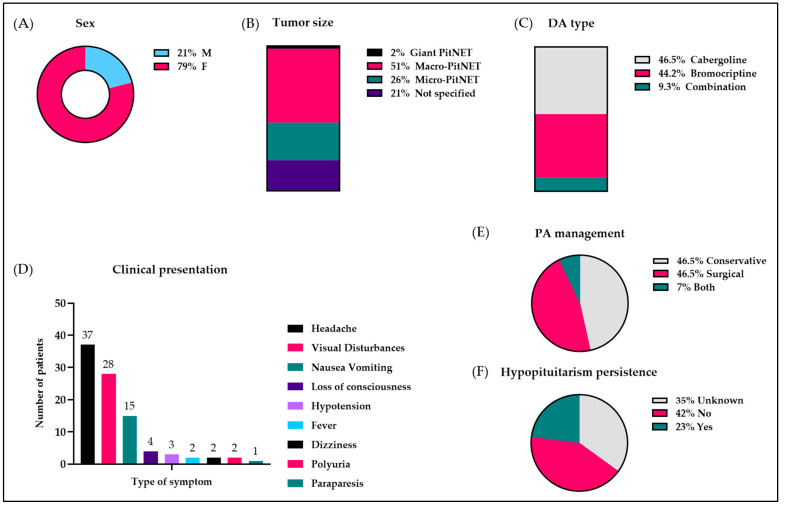
Clinical presentation and outcomes of pituitary apoplexy (PA) in patients taking dopamine agonists. (**A**) Sex; (**B**) tumor size; (**C**) dopamine agonist (DA) that was used; (**D**) clinical presentation; (**E**) management of pituitary apoplexy; (**F**) hypopituitarism persistence.

In total, we identified 43 cases of PA associated with the use of DAs that were published between 1979 and 2025. The mean age was 33 years (range 15–57), 79% (34/43) of patients were female, while 21% (9/43) were male. Among the female gender, 24 out of 34 women (56%) developed apoplexy during pregnancy. The initial tumor size was reported in 34 patients, and 22 out of 34 cases (65%) were macro-PitNETs, while 11 out of 34 (32%) were micro-PitNETs, and there was 1 giant PitNET (3%). In terms of hormonal activity, 37 out of 43 cases (86%) were classified as PRL-secreting PitNETs, 3 out of 43 (7%) were classified as GH-secreting PitNETs, 1 out of 43 (2.3%) was a mixed PRL/GH-secreting PitNET, and 1 out of 43 (2.3%) was a TSH-secreting PitNET. Cabergoline was used in 20 out of 43 patients (46.5%), and the dose ranged from 0.25 to 4.5 mg/week. Bromocriptine was used in 19 out of 43 patients (44.2%), and the dose ranged from 5 to 10 mg/day; the remaining 4 patients (9.3%) were treated with both. Time to onset of PA ranged from 2 h [21] to 3.5 years [22] or even more than 8 years in one case [23]. Table 2 and Figure 3 report patients’ characteristics, initial MRI size, tumor type, type of DA used, its dose, and length of treatment before PA.

As summarized in Figure 3, with respect to the clinical presentation, the main symptoms reported by the patients included headache (37/43), visual deficits (28/43), and nausea and vomiting (15/43). Of note, a headache is generally the most prominent, as well as the initial symptom of PA, and it should prompt further analyses in order to confirm or exclude PA. However, in case these exams turn out negative, like in our case, it is important to closely monitor the patient, as PA can be subacute, with slow development of endocrine dysfunction [3]. Regarding the management of PA, 46.5% of cases (20/43 patients) were managed surgically, 46.5% (20/43 patients) were managed conservatively, and 7% (3/43 patients) were managed conservatively but ended up being surgically treated. In the end, PA led to permanent hypopituitarism in 23% of patients (10/43), whereas in 42% of patients (18/43), pituitary function recovered; no data are available for the remaining patients.

## 4. Discussion

PA is a rare endocrine emergency characterized by the sudden onset of headache and visual disturbances, which can be associated with decreased consciousness and hypopituitarism, due to the hemorrhage or infarction of the pituitary gland. Its prevalence is estimated to be about 6.2 cases per 100,000 people [51] and its incidence is about 0.17 cases per 100,000 people per year [52]. In a population study carried out in the United Kingdom, PA occurred in 7.9% of all PitNETs [51], while other studies have reported that PA occurred in a percentage ranging between 2 and 12% of patients with all types of PitNETs [3]. It is estimated that PA may be the presenting symptom of non-functioning PitNETs in 11% of cases [52].

There are several mechanisms/circumstances that have been implicated in the development of this condition [4]. First, the presence of a PitNET, where there can be either an intralesional hemorrhage or a mismatch between blood flow demand and supply, causing ischemic necrosis. Second, low blood supply can lead to pituitary ischemia, such as in the case of hypotension during childbirth. Not surprisingly, pregnancy is a well-known risk factor for this occurrence [53]. Consistent with this concept, in our literature review, most patients with PA were female, because half of them were pregnant women. Third, additional risk factors include pituitary hormonal stimulation (in case of dynamic tests as well as treatment with GnRH agonists or DAs), coagulation disorders/anticoagulation therapy, and/or other therapies [3,4]. Nevertheless, the main cause of PA remains the hemorrhage or infarction of a PitNET.

PA can involve any histological pituitary tumor, but it is more common in non-functioning PitNETs [3,5,6] and prolactinomas [7,8]. In non-functioning PitNETs, the prevalence of PA varies between 3.7% and 21% [54,55,56]. The exact reason for the higher incidence of PA in patients with non-functioning PitNETs is not well understood [57]. Nevertheless, it is believed that, due to their non-secretory nature, these tumors become symptomatic at a later stage, which allows them to reach a greater size by the time of diagnosis, whereby they are more prone to infarction/hemorrhage [58]. In a recent systematic literature review including seven studies with 4937 participants, Kajal et al. demonstrated that, apart from pregnancy, the size and the non-functioning status of the tumor were the only significant factors contributing independently toward an apoplectic event [59]. With respect to PRL-secreting PitNETs, the prevalence of PA is roughly 6.8%, with a higher rate in macro- (20.3%) as compared to micro-PitNETs (3.1%) [60].

DAs, namely bromocriptine and/or cabergoline, are one of the possible precipitating factors of PA [3]. These agents represent the first-line therapy for PRL-secreting PitNETs, as they bind to D2Rs that are highly expressed in lactotroph cells, leading to a reduction in prolactin secretion and a decrease in lesion size [11]. In PRL-secreting PitNETs, DAs promote tumor regression/shrinkage due to the reduction of lactotroph cell size and to degenerative and necrotic changes in tumor cells, which may be followed by subsequent replacement fibrosis [33]. Consistent with these concepts, in our literature review, we found 43 cases of pituitary apoplexy associated with the use of DAs (cabergoline and bromocriptine), and the majority of them were PRL-secreting (90%) PitNETs, while the remaining PitNETs were secreting GH or TSH.

By contrast, here we present a case of PA in a non-functioning PitNET during cabergoline therapy. Although cabergoline is indicated in the treatment of PRL-secreting PitNETs, it can also be considered in non-functioning PitNETs as an off-label treatment. A few studies have shown that cabergoline can induce tumor shrinkage or restrain growth in non-functioning PitNETs [12,61]. In particular, rates of tumor shrinkage range between 20 and 38% and rates of stabilization range between 48 and 51% [12,61].

Although association does not mean causality, in our case, cabergoline might have promoted PA. The question that remains unanswered is how cabergoline might have caused it in a non-functioning PitNET. Based on the 2017 classification by the World Health Organization [62], clinically silent PitNETs, which do not show hormone hypersecretion and are considered non-functioning, may express one of the three main differentiation factors or none. These differentiating transcription factors include PIT1 for somatotroph, lactotroph, and thyrotroph cells, TPIT for corticotroph cells, and SF1 for gonadotroph cells. Silent/non-functioning PitNETs may include silent PIT1, silent TPIT, silent SF1 tumors, as well as null-cell (NULL) PitNETs, whose transcription factor remains unknown. It has been recently shown that D2R is significantly upregulated in PRL-secreting PitNETs, as well as in silent SF1 PitNETs and NULL PitNETs, and to a lower extent also in silent PIT1 tumors [63].

Having said that, although only immunohistochemical testing would provide a definitive answer to our case (which we do not have as the patient was managed conservatively and did not undergo surgery), we can formulate a few hypotheses. One potential explanation of our case is that the patient had a silent, immature PIT1 lineage tumor, which can be found in young individuals [64]. Immature PIT1 lineage tumors are characterized by poorly differentiated cells and may present with features of hormonal hypersecretion, including acromegaly, hyperprolactinemia, or TSH excess, but they may be clinically silent as well [65]. Pellegrini-Bouiller et al. analyzed PIT1 and D2R gene expression in a series of 30 human lactotroph and somatotroph pituitary tumors, identifying a positive correlation between PIT1 mRNA and D2R mRNA levels, which in turn is associated with therapeutic responsiveness to DAs [66]. Consistent with the concept that silent PIT1 lineage tumors may express different receptors, last year, Shiraishi et al. reported a case of PA occurring in a non-functioning PitNET after the administration of lanreotide for the treatment of a pancreatic NET. The patient underwent transsphenoidal surgery, and final pathology showed that the tumor was a plurihormonal adenoma of PIT1 lineage, immunopositive for GH and PRL, with a strong expression of somatostatin receptor subtype 2 [67].

Otherwise, our patient might have had a silent SF1 or a NULL PitNET, which represent 70–75% of non-functioning PitNETs and the subtype with the highest expression of D2Rs after PRL-secreting PitNETs [68]. Tumoral D2R expression represents one of the reasons supporting the use of DAs in non-functioning PitNETs [12], and its abundance seems to correlate with the clinical response [69]. Interestingly, none of the studies evaluating the efficacy of DAs in non-functioning PitNETs reported any PA following cabergoline treatment.

To our knowledge, this is the first report of a case of PA in a non-functioning PitNET during cabergoline therapy. This case confirms cabergoline efficacy in non-functioning PitNETs, and it sheds light on a possible complication of its use. Nevertheless, from our point of view, cabergoline should not be avoided in non-functioning PitNETs, but caution should be taken in large tumors, as size is a risk factor for PA. Patients should be closely monitored, keeping in mind that the most common presenting symptoms of PA are headache and visual deficits, and that almost half of the patients may present subacutely and non-acutely with symptoms developing over several days [70].

In the end, in our case, the patient was managed conservatively and had a complete recovery of pituitary function. Patients with PA frequently experience irreversible pituitary damage, with full recovery of pituitary function reported in only 5–37% of cases [3,4]. However, a recent comprehensive analysis by Ragate et al. involving 93 cases of functioning and non-functioning PitNETs demonstrated comparable rates of hormonal axis recovery between surgically and conservatively managed patients [70]. These figures are consistent with the 42% (18/43) rate of recovery that we found in our literature review. Therefore, endocrine outcomes should not be considered a primary criterion when choosing between surgical and conservative management, as both approaches appear to have a similar impact on endocrine prognosis.

## 5. Conclusions

In the literature, we found 43 cases of PA associated with the use of DA. All the patients had secreting tumors. By contrast, here we present a case of PA in a non-functioning PitNET during cabergoline therapy. Our patient was managed conservatively, and endocrine function recovered spontaneously. In our case, cabergoline might have promoted PA, which is consistent with the reported efficacy of cabergoline in inducing tumor shrinkage of non-functioning PitNETs that express D2Rs, including silent PIT1 and SF1 or NULL tumors. Our case confirms cabergoline efficacy in non-functioning PitNETs and sheds light on a possible complication of its use. Patients, particularly those with large tumors, should be closely monitored for this occurrence.

## Figures and Tables

**Table 1:** Hormonal levels in the case report.

	Basal	PA	2 wks After PA	2 mo After PA	4 mo After PA	6 mo After PA	rr
PRL (ng/mL)	8.9	<0.094	-	-	-	-	2.6–13.0
LH (mIU/L)	-	0.6	1.0	2.5	-	-	1.5–9.2
FSH (mIU/L)	1.80	1.1	0.80	1.7	-	-	1.00–14.00
T (ng/mL)	3.5	<0.025	0.5	4.9	4.5		1.8–7.8
IGF-1 (ng/mL)	153	120	-	87	-	-	63.40–223.00
TSH (µIU/mL)	1.76	0.09	0.28	0.61	0.54	1.48	0.40–4.00
fT4 (pg/mL)	7.5	9.5	7 (LT4)	7.6 (LT4)	8.8 (LT4)	6.9	5.6–12.0
ACTH (pg/mL)	22	8.8	10.3	-	14.3	-	4.2–48.8
Cortisol (nmol/L)	145	46.9	236	229	231	350	185–624

ACTH, adrenocorticotropic hormone; FSH, follicle stimulating hormone; fT4, free thyroxine; IGF-1, insulin-like growth factor-1; LH, luteinizing hormone; LT4, levothyroxine; mo, month; PA, pituitary apoplexy; PRL, prolactin; rr, reference range; T, testosterone; TSH, thyroid stimulating hormone; wks, week; “-”, no data.

**Table 2:** Case reports of patients with PA associated with the use of DAs.

Reference	Age	Sex	Pregnancy	Tumor Size (cm)	Tumor Type	DA Type	Dose	Time of Onset
Lamberts, 1979 [24]	30	F	Yes	-	PRL	B	-	-
Yamaji, 1981 [25]	46	M	No	-	GH	B	7.5 mg/d	2 yrs
Yamaji, 1981 [25]	55	F	No	-	GH	B	7.5 mg/d	6 mo
Alhajje, 1985 [26]	50	F	No	-	GH	B	5 mg/d	10 d
O’Donovan, 1986 [27]	37	F	Yes	>1	PRL	B	-	-
Shirataki, 1988 [21]	50	F	No	-	GH, PRL	B	2.5 mg/once	2 hrs
Freeman, 1992 [28]	22	F	Yes	>1	PRL	B	-	-
Pinto, 1998 [29]	15	F	No	2.5 × 2 × 2	PRL	B	10 mg/d	6 mo
Vella, 2001 [30]	30	M	No	>1	PRL	C	0.5 mg/wk	5 mo
Gondim, 2003 [31]	29	F	Yes	0.3 × 0.4	PRL	B	5 mg/d	-
Knoepfelmacher, 2004 [32]	17	M	No	2.2 × 2 × 2	PRL	B + C	5 mg/d + 1.5 mg/wk	1 yr
Balarini Lima, 2008 [33]	57	M	No	9.5 × 7.6 × 6.5	PRL	C	3.5 mg/wk	1 mo
Balarini Lima, 2008 [33]	27	M	No	>1	PRL	C	1.5 mg/wk	3 mo
Balarini Lima, 2008 [33]	15	F	No	>1	PRL	C	1.5 mg/wk	8 mo
Monden, 2008 [34]	54	F	No	3.5 × 3.0 × 2.0	GH, TSH	B	2.5 mg/once	24 hrs
Parihar, 2009 [35]	22	F	Yes	>1	PRL	B	-	-
Ginath, 2010 [36]	31	F	-	-	PRL	B	-	-
Couture, 2012 [37]	37	F	Yes	0.7 × 0.7	PRL	C	0.5 mg/wk	-
Witek, 2012 [38]	25	F	Yes	-	PRL	B	-	-
Janssen, 2012 [39]	27	F	Yes	1.95	PRL	B	5 mg/d	-
Al-Sharafi, 2013 [23]	32	F	No	2.1 × 2.8	PRL	(B) + C	4.5 mg/wk	(8 yrs) + 1 yr
Chng, 2013 [17]	20	M	No	2.5 × 1.7 × 2.5	PRL	C	0.5 mg/wk	1.5 mo
Hayes, 2014 [40]	41	F	Yes	0.6 × 0.9	PRL	C	0.5 mg/wk	-
Tandon, 2014 [41]	27	F	Yes	-	PRL	B	-	-
De Ycaza, 2015 [42]	26	F	Yes	1.9 × 1.3	PRL	B + C	5 mg/d + 0.5 mg/wk	-
Grand’Maison, 2015 [18]	30	F	Yes	1.3 × 1.7 × 1.0	PRL	C	0.5 mg/wk	1 mo
Grand’Maison, 2015 [18]	37	F	Yes	0.7 × 0.7	-	C	0.5 mg/wk	1.5 yrs
Galvão, 2016 [43]	30	F	Yes	<1	PRL	B	5 mg/d	2 mo
Galvão, 2016 [43]	-	F	Yes	<1	PRL	B	5 mg/d	2 mo
Margari, 2016 [44]	52	F	No	>6	PRL	C	-	1 wk + 3 mo
Annamalai, 2017 [45]	25	F	Yes	0.4	PRL	C	0.25 mg/wk	3 mo
Aydin, 2018 [46]	49	M	No	2.1 × 2 × 2.1	PRL	C	1 mg/wk	4 mo
Ghadirian, 2018 [47]	34	M	No	>1	PRL	C	2 mg/wk	1 yr
Oguz, 2020 [48]	22	F	Yes	>1	PRL	C	-	-
Khaldi, 2021 [22]	30	F	Yes	4 × 3.6 × 3	PRL	C	3 mg/wk	3.5 yrs
Kuhn, 2021 [19]	31	F	Yes	0.7	PRL	C	0.5 mg/wk	-
Khun, 2021 [19]	21	F	Yes	0.7	PRL	B	5 mg/d	-
Khun, 2021 [19]	32	F	Yes	1	PRL	C	0.5 mg/wk	-
Khun, 2021 [19]	23	F	Yes	0.5	PRL	C	0.5 mg/wk	-
Khun, 2021 [19]	25	F	Yes	<1	PRL	C	-	-
Jamal, 2024 [49]	45	F	No	5.0 × 4.2 × 2.8	PRL	C	1 mg/wk	-
Cholekho, 2024 [50]	51	M	No	3.2 × 2.6 × 2.1	PRL	B	5 mg/d	1 mo
Introini, 2025 [20]	29	F	Yes	0.3 × 0.5 × 0.4	PRL	B + C	5 mg/d + 1 mg/wk	-

B = bromocriptine, C = cabergoline, d = day, F = female, GH = growth hormone, hr = hour, M = male, mo = month, PRL = prolactin, TSH = thyroid stimulating hormone, yr = year, wk = week, - = no data.

## Data Availability

No new data were created or analyzed in this study.
